# Quality Evolution of Pu-Erh Raw Tea over 1–22 Years of Aging

**DOI:** 10.3390/foods15193448

**Published:** 2026-09-27

**Authors:** Miao Zhou, Xinya Chen, Dianrong Ruan, Xin Fang, Jun Gao, Changmin Cai, Yujie Wu, Shijie Lu, Zuzhen Wang, Xiujuan Deng, Baijuan Wang

**Affiliations:** 1 College of Tea Science, Yunnan Agricultural University, Kunming 650201, China; 18724979985@163.com (M.Z.); chenxinya128@163.com (X.C.);; 2 Yunnan Liuda Chashan Tea Industry Co., Ltd., Kunming 650224, China

**Keywords:** raw Pu-erh tea, storage duration, aging stages, ROAV

## Abstract

Long-term aging transforms the sensory quality of raw Pu-erh tea, but the temporal coordination between taste-related and aroma-active compounds remains unclear. Here, raw Pu-erh teas aged for 1–22 years, produced in the same region and stored under consistent conditions, were characterized using sensory evaluation, targeted analysis of non-volatile constituents, headspace solid-phase microextraction coupled with gas chromatography–mass spectrometry, relative odor activity values (ROAVs), and multivariate statistics. Aging shifted the sensory profile from fresh, strong, and astringent to mellow and aged, accompanied by a change in liquor color from yellow-green to orange-red. Non-volatile profiles resolved four aging stages (1–4, 6–10, 14–18, and 19–22 years), whereas key aroma-active compounds resolved three stages (1–10, 14–19, and 20–22 years). Tea polyphenols, catechins, and free amino acids generally declined, while theabrownins, thearubigins, gallic acid, and selected flavonoids accumulated. Among 523 detected volatile compounds, 39 exhibited ROAVs ≥ 1; β-ionone and (E)-2-nonenal formed a persistent floral–woody aroma backbone, while stage-specific changes increasingly favored resinous, camphoraceous, roasted, and aged notes. Notably, differences in non-volatile composition weakened after 14 years, whereas aroma profiles continued to reorganize. This asynchronous evolution constitutes the central finding of this study, demonstrating that taste-related composition approaches relative stability before aroma maturation is complete and providing a chemical basis for aging-stage classification and quality evaluation.

## 1. Introduction

Raw Pu-erh Tea is a compressed tea produced from sun-dried green tea made from Camellia sinensis var. assamica leaves in Yunnan Province, China, which is then subjected to steaming and pressing. It is a traditional Chinese tea characterized by pronounced geographical specificity and aging potential [1]. Unlike other tea types, Raw Pu-erh Tea undergoes continuous post-fermentation transformations under suitable storage conditions, during which its quality evolves dynamically with prolonged storage, ultimately developing the distinctive characteristic of “improving aroma with age” [2]. This unique property endows Raw Pu-erh Tea not only with drinking value but also with attributes of collection and investment, and the market demand for high-quality aged Raw Pu-erh Tea has continued to increase in recent years [3].

The aging and storage (post-ripening) of Raw Pu-erh Tea is a complex process driven by the synergistic interactions of multiple factors, including microbial metabolism, enzymatic reactions, hydrothermal effects, and oxidation processes [4]. Previous studies have demonstrated that storage environmental conditions (e.g., temperature, humidity, oxygen availability, and light exposure) play a decisive role in determining the rate of aging and the direction of quality evolution [5,6].

In recent years, a substantial body of research has been conducted both domestically and internationally on quality changes in Raw Pu-erh Tea during storage. Zhou et al. [6] investigated the quality evolution of Raw Pu-erh Tea aged 3–20 years and reported that sensory quality improved after 10 years of storage; however, it began to decline after 18 years. Cluster analysis further classified the samples into five distinct groups. Liu et al. [7] systematically analyzed Raw Pu-erh Tea from the same production area but different storage years and found that gallic acid increased significantly, while caffeine and catechins decreased. Among volatile-related components, hydrocarbons accounted for the highest proportion of aroma compounds, with more pronounced changes observed after more than 5 years of storage.

In terms of non-volatile constituents, water extract content exhibited a decreasing–increasing trend, tea polyphenols showed a continuous decline, and soluble sugars first decreased and then increased. Meanwhile, theabrownins and thearubigins gradually accumulated, and flavonoid compounds (e.g., rutin and quercetin) increased significantly with aging time [8,9]. Ester-type catechins (such as EGCG and ECG) generally decreased during aging; however, some studies have reported secondary fluctuations during long-term storage [10]. The total free amino acid content typically decreases with prolonged storage, but an increase has been observed in certain long-aged samples, which may be associated with microbial-mediated proteolysis [11].

In terms of aroma composition, Zhao et al. [12] employed a combined volatilomics and microbiomics approach and found that the aroma profile of Raw Pu-erh Tea shifted from floral and fruity notes toward aged and woody characteristics during storage. β-ionone and (E)-2-nonenal were identified as key aroma backbone compounds persisting throughout the entire aging process, while 1,2,3-trimethoxybenzene and 1,2,4-trimethoxybenzene accumulated significantly during long-term storage.

Xu et al. [13] compared the effects of dry–cool and warm–humid storage environments on the aroma profile of Raw Pu-erh Tea and reported that warm–humid conditions significantly increased the levels of methoxybenzene compounds, thereby contributing to aged and woody sensory characteristics.

Yang et al. [14] integrated hyperspectral imaging with machine learning and microbiome analysis to successfully construct a non-destructive model for distinguishing storage age of Raw Pu-erh Tea, and further revealed the association between core microbial taxa, such as Bacillus, and chemical transformation processes during aging.

Despite the substantial progress achieved in this field, several limitations remain in current studies. First, most investigations have focused on short-term (1–5 years) or medium-term (6–10 years) storage samples, while systematic research on long-term aging (over 10 years) and especially ultra-long storage exceeding 20 years remains relatively limited [12]. Second, due to considerable variability in raw material origin, processing techniques, and storage conditions of Pu-erh tea, there are substantial differences in sample backgrounds among studies, and systematic longitudinal tracking of sequential-year samples from the same production area under consistent storage conditions is still lacking [15]. Third, existing studies have predominantly focused on either non-volatile or volatile constituents separately, and comprehensive analyses of their coordinated and interactive evolution remain insufficient [16]. Finally, the correspondence between clustering-based stage division of volatile aroma compounds and that of non-volatile constituents, as well as the stability of key “aroma backbone” compounds during long-term aging, still requires further clarification.

Establishing a continuous sample set covering 1–22 years of storage enables a systematic characterization of the dynamic trajectories of sensory quality and chemical composition during the long-term aging of raw Pu-erh tea, while facilitating the identification of stage-specific changes and critical transition points that may be overlooked when samples from only discrete storage years are examined. On this basis, quality markers exhibiting stable time-dependent responses can be further screened, and associations between storage duration and chemical characteristics can be established, thereby providing a more systematic data foundation for the classification of aging stages, quality control, and authentication of storage age in Pu-erh tea.

Raw Pu-erh Tea samples produced between 2002 and 2023 and stored in Yiwu, Xishuangbanna, Yunnan Province were selected as experimental materials. A comprehensive approach integrating sensory evaluation, physicochemical analysis, high-performance liquid chromatography (HPLC), and headspace solid-phase microextraction coupled with gas chromatography–mass spectrometry (HS-SPME-GC-MS), combined with multivariate statistical analysis and odor activity value (OAV/ROAV) methods, was employed to systematically investigate the quality characteristics of Raw Pu-erh Tea and the dynamic changes in non-volatile and volatile compounds during 1–22 years of storage.

This study aimed to: (1) elucidate the stage-dependent quality evolution characteristics of Raw Pu-erh Tea during long-term storage; (2) identify key differential physicochemical indices and aroma-active compounds; and (3) reveal the coordinated transformation relationships between non-volatile and volatile constituents. The findings are expected to provide a theoretical basis for quality control, optimal drinking period determination, and age authentication of Raw Pu-erh Tea.

## 2. Materials and Methods

### 2.1. Preparation of Tea Samples

This study selected Raw Pu-erh Tea samples produced over a continuous 22-year period (2002–2023) from the same origin (Yiwu, Yunnan Province) and stored under identical conditions. The tea samples were stored as compressed tea cakes, with each cake individually wrapped in food-grade cotton paper. The samples were placed on shelves with sufficient clearance from the floor and walls. During long-term storage, the ambient temperature was maintained at approximately 20–30 °C, and the relative humidity was controlled at 40–50%. The warehouse was kept dry, clean, moderately ventilated, and free from odors. The samples were protected from direct sunlight and stored separately from materials that could generate odors, contain hazardous substances, or cause cross-contamination. The standardized warehouse implemented year-round environmental monitoring, regular sensory evaluations of brewed tea, and annual third-party sampling and testing. Samples were provided by Yunnan Six Famous Tea Mountains Tea Co., Ltd. All raw materials were derived from local Yunnan large-leaf population cultivars, using one bud with two to three leaves as fresh leaf material.

The processing procedure included the following steps: fresh leaf harvesting, withering, fixation (kill-green), rolling, sun-drying, and compression. The Raw Pu-erh Tea samples were stored in a dry and well-ventilated warehouse under natural storage conditions. Detailed sample information is presented in Table 1.

### 2.2. Sensory Evaluation

#### 2.2.1. Sensory Evaluation

Sensory evaluation was performed according to GB/T 23776-2018 “Methodology for Sensory Evaluation of Tea” [17] and GB/T 14487-2017 “Terms of Tea Sensory Evaluation” [18] by five trained professional tea assessors. Before formal evaluation, the panelists received calibration training using representative raw Pu-erh tea samples with different storage ages to harmonize the interpretation of sensory descriptors and scoring criteria. Samples were coded with random three-digit numbers and evaluated under blinded conditions in a sensory evaluation room free of interfering odors. The serving order was randomized, and warm water was provided for palate cleansing between samples. Each sample was evaluated in three independent sessions, and the final sensory score was calculated as the mean value. Disagreements among assessors were discussed only after independent scoring, and the final descriptive terms were recorded according to the consensus of trained panelists.

The evaluation panel consisted of five trained assessors from the College of Tea Science, Yunnan Agricultural University, all of whom held professional tea taster certification. For each sample, 3.0 g of tea was placed in a professional tasting cup, followed by the addition of 150 mL of boiling water. The infusion was steeped for 5 min and brewed twice. Sensory attributes including appearance, aroma, liquor color, taste, and infused leaf were evaluated and recorded.

The total sensory score was calculated as follows: appearance (20%), liquor color (10%), aroma (30%), taste (35%), and infused leaf (5%).

#### 2.2.2. Analysis of Nonvolatile Compounds

Sample pretreatment and physicochemical analyses: All tea samples were ground after being labeled according to their corresponding codes, passed through a 40-mesh sieve, and then stored in airtight containers at −20 °C for further analysis.

The contents of tea polyphenols were determined according to GB/T 8313–2018 “Determination of Tea Polyphenols and Catechins in Tea” [19], including catechin (C), epicatechin (EC), epigallocatechin (EGC), epicatechin gallate (ECG), epigallocatechin gallate (EGCG), gallocatechi (GC), and gallocatechin gallate (GCG); free amino acids(FAA)were measured following GB/T 8314–2013 “Determination of Total Free Amino Acids in Tea” [20]; soluble sugars were quantified using the anthrone–sulfuric acid colorimetric method; and water extract content was determined according to GB/T 8305–2013 “Determination of Water Extracts in Tea” [21]. Theabrownin (TB), thearubigin (TR), and theaflavin (TF) contents were determined using a systematic analytical method [22].

The contents of catechins, flavonoids, and purine alkaloids were determined by high-performance liquid chromatography (HPLC) based on the method described by Nian et al. [23]. All samples were extracted and analyzed in triplicate.

#### 2.2.3. Determination of Volatile Components

Volatile compounds in the samples were isolated and identified using headspace solid-phase microextraction coupled with gas chromatography–mass spectrometry (HS-SPME-GC-MS).

(1) Headspace solid-phase microextraction (HS-SPME)

A 1.00 g portion of each sample was placed in a 20 mL headspace vial, followed by the addition of 1.00 µg ethyl decanoate (decanoic acid ethyl ester) as an internal standard and 6 mL of boiling water. The relative concentrations of volatile compounds were estimated by a semi-quantitative method using ethyl decanoate as a single internal standard. Therefore, the results were mainly used for comparative analysis among samples rather than absolute quantification of different compound classes. It should be noted that the use of a single internal standard may not fully account for differences in extraction efficiency and MS response among different volatile compound classes. Future studies using class-specific or isotope-labelled internal standards would further improve the quantitative accuracy of volatile compound analysis. The vial was immediately sealed. Extraction was performed using a CTC autosampler under the following conditions: equilibration at 60 °C for 10 min, extraction with a 65 µm polydimethylsiloxane/divinylbenzene (PDMS/DVB; Supelco, Bellefonte, PA, USA; catalogue No. 57293-U) fiber at 60 °C for 30 min with stirring at 250 rpm. Thermal desorption was carried out in the GC inlet at 230 °C for 5 min.

(2) GC-MS conditions

Chromatographic separation was carried out using an Agilent J&W DB-WAX capillary column (30 m × 0.25 mm i.d. × 0.25 μm film thickness; Agilent Technologies (Santa Clara, CA, USA); catalogue No. 122-7032). High-purity helium (≥99.999%) was used as the carrier gas at a constant flow rate of 1.0 mL min^−1^. The initial oven temperature was maintained at 50 °C for 5 min, increased to 230 °C at 6 °C min^−1^, and then maintained at 230 °C for 7 min. Mass spectrometric conditions were as follows: electron impact (EI) ionization mode; ion source temperature, 230 °C; interface temperature, 230 °C; and quadrupole temperature, 150 °C.

(3) Qualitative and quantitative analysis

All volatile compounds were initially identified by matching their mass spectra with the National Institute of Standards and Technology (NIST 17) library. Identification was further confirmed by comparison of retention indices (RI). Relative concentrations of individual compounds were calculated using an internal standard semi-quantitative method. The relative odor activity value (ROAV) of each compound was calculated to evaluate its contribution to overall aroma. The ROAV was determined using the following equation [24]:

$$ROAV=\frac{C_{A}}{C_{max}}\times \frac{T_{max}}{T_{A}}\times 100$$

where *C_A_* and *T_A_* represent the relative concentration and odor threshold of any volatile compound, respectively (%), and *C_max_* and *T_max_* represent the relative concentration and odor threshold of the compound with the highest odor contribution, respectively (%).

#### 2.2.4. Statistical Analysis

Data standardization and preliminary data processing were performed using Microsoft Excel 2021. All measurements were conducted in triplicate, and the results are expressed as mean ± standard deviation (x ± SD). For sensory evaluation, the scores from five trained panelists were averaged for each sample. Prior to significance analysis, the normality of the data and homogeneity of variance were evaluated using the Shapiro–Wilk test and Levene’s test, respectively. When the assumptions of normality and homogeneity of variance were satisfied, differences among samples were analyzed by one-way analysis of variance (ANOVA), followed by Tukey’s honestly significant difference (HSD) post hoc test. When these assumptions were not met, the Kruskal–Wallis test was used, followed by Dunn’s multiple comparison test. Statistical significance was defined as *p* < 0.05.

Multivariate statistical analyses, including principal component analysis (PCA) and orthogonal partial least squares-discriminant analysis (OPLS-DA), were performed using SIMCA 14.1 software. Prior to multivariate analysis, the data were normalized and scaled to reduce the influence of variable magnitude. The quality and reliability of the OPLS-DA models were evaluated using R^2^X, R^2^Y, and Q^2^ values. Model robustness was further assessed by cross-validation and permutation testing. A 200-time permutation test was performed to evaluate whether the OPLS-DA model was overfitted. Models were considered reliable when they showed acceptable explanatory and predictive ability, with Q^2^ > 0.5 and the intercept of the permuted Q^2^ regression line below zero. Variables with variable importance in projection (VIP) values > 1.0 and *p* < 0.05 were considered important differential variables. Data visualization was conducted using TBtools V2.1 and Chiplot (www.chiplot.online).

## 3. Results and Analysis

### 3.1. Sensory Flavor Evolution of Raw Pu-Erh Tea Across Different Storage Years

Sensory evaluation results indicated that Raw Pu-erh Tea samples stored for 1–22 years exhibited an overall quality evolution from a strong, fresh, and brisk profile toward an aged and mellow character with increasing storage time (Figure 1). In terms of appearance, samples aged 1–7 years showed a greenish-brown color, whereas samples stored for more than 8 years gradually developed a green-brown with reddish tones. Samples stored for 2, 3, 7, 16, and 18 years received the highest appearance scores (92 points).

Regarding aroma, the 1-year sample was dominated by floral notes; samples aged 2–4 years mainly exhibited a clean aroma; samples aged 6 and 7 years showed a combination of clean and woody notes; and samples aged 8 years and above gradually developed aged aroma characteristics. However, the intensity of aged aroma decreased with further increases in storage time. The samples stored for 1, 6, 7, and 17 years obtained the highest aroma scores (94 points).

In terms of taste, samples aged 1–3 years were characterized by strong intensity with pronounced bitterness and astringency. From 4 to 10 years, sweetness began to emerge, although bitterness remained evident; notably, astringency increased again with a slight sour note observed in the 9–10-year samples. The 15-year sample exhibited a sweet, mellow taste with enhanced aftertaste and reduced astringency. Samples aged 17–22 years showed a relatively flat taste profile with decreased mellowness. The samples stored for 1, 7, 15, and 17 years achieved the highest taste-related scores (90 points).

For infused leaves, samples aged 1–7 years were predominantly yellow-green; those aged 8–10 years were mainly greenish-brown; samples aged 14–20 years were primarily brownish-green; and samples stored for more than 20 years again exhibited a greenish-brown color.

Overall, the highest comprehensive sensory score was observed in the 7-year sample (92 points), followed by the 1-year sample (91.5 points).

In summary, the sensory evaluation demonstrated a clear trend in Raw Pu-erh Tea during 1–22 years of storage, transitioning from a strong and fresh profile to an aged and mellow character. Samples aged 1–7 years were characterized by greenish-brown appearance, fresh or floral aroma, and strong, bitter, and brisk taste. Samples aged 8–15 years exhibited a gradual development of aged aroma, sweet and mellow taste with reduced bitterness and astringency, and greenish-brown to brownish-green infused leaves. Samples aged 16–22 years showed a flatter taste profile with reduced mellowness. Overall, the highest sensory quality was observed in the 7-year sample, followed by the 1-year sample.

### 3.2. Analysis of Non-Volatile Compounds in Raw Pu-Erh Tea Across Different Storage Years

The taste of tea infusion is the combined result of multiple water-soluble taste-active compounds acting on taste receptors, and is mainly influenced by the content and proportional relationships of polyphenols, alkaloids, amino acids, sugars, and organic acids. With the extension of storage time, the major quality-related components of raw Pu-erh tea showed obvious dynamic changes (Figure 2). The water extract content generally remained at a relatively high level, increasing from 44.18% in the 1-year sample to 50.71% in the 14-year sample, and then gradually decreasing to 40.59% in the 21-year sample. This indicates that soluble substances remained relatively abundant during long-term storage, while their decrease in the later stage may be attributed to oxidation, polymerization, or degradation. The free amino acid content showed an overall decreasing trend with storage time, maintaining a relatively high level during 1–4 years of storage (4.70–5.05%) and decreasing to approximately 1% during 14–18 years. This suggests that amino acids may participate in oxidation, degradation, and Maillard reactions, which is consistent with the weakening of fresh taste during aging.

The soluble sugar content was relatively stable in the early and middle storage stages, reaching a relatively high level of 5.13–5.48% during 2–4 years and peaking at 5.60% in the 14-year sample. However, it decreased markedly in the later stage, especially to 1.97% in the 22-year sample, indicating that sugars may be continuously consumed or involved in flavor transformation during long-term storage. The tea polyphenol content showed an overall decreasing trend, gradually decreasing from 23.44% in the 3-year sample to 20.77% in the 22-year sample. This reflects the oxidation, polymerization, and transformation of tea polyphenols during storage, which serves as an important material basis for the transition of raw Pu-erh tea from bitter, astringent, and fresh characteristics to a mellow taste and aged aroma.

Theaflavins, thearubigins, and theabrownins exhibited different changing patterns. The theaflavin (TF) content remained at a relatively low level in the early stage (0.02–0.04%) and increased markedly to 0.08–0.09% during 17–20 years, indicating the accumulation of polyphenol oxidation products in the middle and late storage stages. The thearubigin (TR) content reached a relatively high level during 14–17 years, with the highest value of 2.36% observed in the 16-year sample, suggesting that this period may be an active stage for color and taste transformation. The theabrownin (TB) content showed an overall increasing trend, rising from 0.62% in the 1-year sample to 1.49% in the 22-year sample, and remained at a relatively high level in the later stage. This indicates that the degree of oxidative polymerization increased with prolonged storage, resulting in a gradual deepening of tea infusion color and more pronounced aging characteristics. Nevertheless, because instrumental chromaticity parameters and a specific browning index were not measured, these pigment changes should be interpreted as indirect chemical evidence of color development rather than as a direct quantitative measurement of browning.

Different catechin components showed distinct changing patterns. The contents of C and CG fluctuated considerably, among which CG increased markedly in the 9-year sample, possibly related to the isomerization or transformation of other catechins. EC, ECG, and EGC showed overall decreasing trends, especially EGC and ECG, which declined more obviously. This suggests that these esterified or non-esterified catechins are more prone to oxidation and degradation or may participate in the formation of polymerized products such as theaflavins, thearubigins, and theabrownins during long-term storage. Overall, the decrease in catechins was consistent with the declining trend of tea polyphenols, further indicating that long-term storage weakened the bitter, astringent, and fresh characteristics of tea and promoted the formation of a mellow taste.

To further elucidate the chemical basis of taste characteristics in Raw Pu-erh Tea across different storage years, cluster analysis was performed on 24 fundamental physicochemical constituents. The results (Figure 3) indicated that all samples could be broadly classified into four major groups, namely YW1 (1–4 years), YW2 (6–10 years), YW3 (14–18 years), and YW4 (19–22 years). This grouping pattern was consistent with the overall sensory evaluation trends observed for aroma and taste evolution.

Meanwhile, to further screen key differential compounds among different storage-year groups, OPLS-DA analysis was performed on the four clusters. Based on the criteria of VIP > 1, FC ≥ 2 or ≤ 0.5, and *p* < 0.05, a total of 11 major differential physicochemical constituents were identified, including free amino acids (FAA), myricetin, epigallocatechin (EGC), luteolin, quercetin (Que), gallic acid (GA), gallocatechin (GC), gallocatechin gallate (GCG), theaflavins (TF), catechin (C), and epigallocatechin gallate (EGCG).

The results (Figure 4) indicated that significant differences were observed between YW1/YW2 and YW3/YW4 groups, whereas only one significantly different compound was detected between YW3 and YW4. This suggests that after more than 14 years of storage, the major physicochemical components of Raw Pu-erh Tea tend to reach a relatively stable state.

The results of differential metabolite analysis indicated that free amino acids (FAA) served as key marker compounds distinguishing early- and late-stage storage samples. FAA levels were significantly higher in YW1 and YW2 than in YW3 and YW4, and the fold-change differences gradually decreased with increasing storage time (FC values: YW1 vs. YW3/YW4 = 7.38 and 3.83; YW2 vs. YW3/YW4 = 5.06 and 2.63), indicating a continuous decline in FAA content during storage.

In contrast, myricetin exhibited an opposite trend, showing significant accumulation in the later storage groups, suggesting that it is an important flavonoid progressively enriched during aging. Theaflavins (TF) and gallic acid (GA) were significantly lower in early storage samples, indicating continuous formation or accumulation during long-term storage (FC values for TF: YW1/YW2 vs. YW4 = 0.40 and 0.41; FC values for GA: YW2 vs. YW3/YW4 = 0.33 and 0.18).

Among catechin components, EGC, GCG, and GC exhibited distinct stage-dependent variation patterns. EGC reached relatively high levels in YW2, whereas GCG and GC were relatively enriched in YW1 and YW2 groups. Overall, with increasing storage duration, the chemical constituents of Raw Pu-erh Tea underwent significant transformation, characterized by the gradual depletion of certain amino acids and catechins, along with the continuous accumulation of flavonoids and oxidative polymerization products. These coordinated changes collectively drive the quality evolution of Raw Pu-erh Tea toward an aged and mellow profile.

The combined results of sensory evaluation and physicochemical analysis demonstrated a pronounced aging pattern in Raw Pu-erh Tea during 1–22 years of storage. With increasing storage time, taste-active compounds associated with freshness, intensity, bitterness, and astringency—such as tea polyphenols, catechins, and free amino acids—were continuously degraded, whereas theaflavins, thearubigins, theabrownins, gallic acid, and certain flavonoids progressively accumulated. These coordinated transformations drove the sensory evolution of the tea from an early stage characterized by strong and fresh attributes, to a mid-stage with a sweet and well-balanced profile, and finally to a late-stage exhibiting aged and mellow characteristics.

Notably, the 8–15 year storage period exhibited a relatively optimal balance among aroma, taste, and chemical composition, corresponding to the highest overall sensory quality. In contrast, long-term storage was associated with excessive depletion of key constituents, resulting in a flatter and less pronounced taste profile.

Overall, the quality formation of Raw Pu-erh Tea is governed by the continuous transformation of freshness-related compounds and the progressive accumulation of oxidative polymerization products. The evolution of chemical composition is highly consistent with changes in sensory quality during storage.

### 3.3. Analysis of Volatile Compounds in Raw Pu-Erh Tea Across Different Storage Years

#### 3.3.1. Analysis of Aroma Compounds in Raw Pu-Erh Tea Across Different Storage Years

Volatile constituents of Raw Pu-erh Tea samples from different storage years were analyzed using HS-SPME/GC-MS. A total of 523 volatile compounds were detected in all the tea samples, which were classified into 13 chemical groups (Figure 5), including ketones (79 compounds), alkenes (71), heterocyclic compounds (70), alcohols (64), esters (58), alkanes (49), aromatic hydrocarbons (46), aldehydes (33), phenols (19), carboxylic acids (11), ethers (10), nitrogen-containing compounds (8), and other compounds (5).

Among all samples, the 2002 sample exhibited the highest number of volatile compounds (112 compounds), whereas the 2021 sample showed the lowest (73 compounds). Overall, the number of detectable volatile compounds increased with prolonged storage, indicating a progressive enrichment and increased complexity of aroma composition. In terms of total relative concentration, the 2017 sample showed the highest value (23.95 mg/g), followed by the 2005 sample (20.02 mg/g). The lowest total concentration was observed in the 2006 sample (4.26 mg/g), followed by the 2009 sample (4.70 mg/g).

Although a wide variety of volatile compounds were detected in tea, only a subset with high odor activity typically plays a dominant role in aroma perception. Based on odor threshold values and aroma descriptors, a total of 145 aroma-active compounds were identified in this study, mainly contributing to fruity, floral, woody, sweet, green, fatty, spicy, nutty, camphor-like, aged, and fresh aroma notes.

Floral, fruity, and woody notes were the dominant aroma types across all storage years (Figure 6). Samples aged 1–7 years were mainly characterized by floral, fruity, and woody aromas. Among them, the 7-year sample exhibited the highest relative contents of floral (4.30 mg/g) and woody (5.65 mg/g) notes, while the 2-year sample showed the highest fruity aroma content (4.82 mg/g). In samples aged 8–14 years, fruity and woody notes remained dominant, while camphor-like aroma began to increase.

For samples aged over 15 years, floral and fruity notes generally decreased, and the overall aroma profile became more balanced. Notably, the 19-year sample exhibited relatively high levels of floral, fruity, and woody notes, as well as the highest sweet aroma intensity. Although aged aroma compounds were present at relatively low levels overall, they reached a maximum in the 21-year sample, which was consistent with the sensory evaluation showing enhanced aged characteristics in the later storage stage. Fatty and sweet notes also accumulated in mid- and late-stage samples, which may be associated with lipid oxidation and Maillard reactions during aging.

Further screening based on ROAVs identified 39 key aroma-active compounds (ROAV ≥ 1.00). The results (Figure 7) showed that β-ionone and (E)-2-nonenal consistently exhibited high aroma activity across the entire storage period, serving as important backbone compounds responsible for floral and woody notes throughout the 1–22-year samples. In addition, the predominant aroma-active compound, defined as the compound with the highest ROAV in each sample, varied among storage years. Hexadecane was the predominant compound in most samples, including the 1-, 4-, 6-, 8-, 9-, and 14–19-year samples, indicating that hydrocarbon-related aroma-active compounds contributed substantially to the aroma profile during these storage periods. In contrast, 2,4,4-trimethyl-2-cyclohexen-1-ol showed the highest ROAVs in the 3-, 7-, and 10-year samples, while β-ionone became the predominant compound in the 2-year sample and in the highly aged 20–22-year samples. These results indicate that the compound with the greatest calculated aroma contribution was not constant across all samples but changed with storage year, suggesting stage-dependent shifts in the dominant aroma-active compounds during long-term storage.

Specifically, β-ionone showed relatively high ROAVs in the 2-year and 20–22-year samples, indicating that violet-like floral and sweet fruity notes were not completely diminished in high-age samples but were re-enhanced at later stages. Similarly, (E)-2-nonenal maintained high ROAVs in the 2-year and 20–22-year samples, suggesting that woody aroma persisted throughout the storage process and became further intensified in later aging stages.

In addition, 2,4,4-trimethyl-2-cyclohexen-1-ol, characterized by honey-like floral notes, showed high aroma activity in the 3-, 7-, and 9-year samples and contributed significantly to aroma perception in these corresponding stages. Terpinen-4-ol increased markedly in the 7- and 9-year samples, imparting spicy and sandalwood-like notes. 4-ethylphenol showed a pronounced increase in the 20-year sample, indicating a particularly strong woody character at this stage. Furthermore, resinous or camphoraceous compounds such as camphene, cedrol, and borneol showed enhanced contributions in high-age samples, collectively strengthening the aged aroma profile.

Overall, the key aroma-active compounds in Raw Pu-erh Tea exhibited the following characteristics with increasing storage time: (1) fundamental backbone aroma compounds persisted throughout the entire aging process; (2) certain characteristic aroma compounds showed stage-dependent enhancement at specific storage periods; and (3) aroma activity in high-aged samples increased again, accompanied by higher compositional complexity.

These results indicate that the aroma evolution of Raw Pu-erh Tea is not a simple unidirectional decline, but rather a dynamic process involving the fluctuation, reorganization, and rebalancing of different classes of aroma compounds during storage.

#### 3.3.2. Multivariate Statistical Analysis of Aroma Compounds in Pu-Erh Raw Tea Across Different Storage Years

Based on cluster analysis of 39 key aroma-active compounds, Raw Pu-erh Tea samples from different storage years were classified into three major groups, namely YW1 (1–10 years), YW2 (14–19 years), and YW3 (20–22 years). This classification indicates that, compared with non-volatile constituents, volatile aroma compounds tend to exhibit a more distinct three-stage differentiation pattern during storage, corresponding to early, mid-to-late, and late high-age stages.

Pairwise comparisons among the three groups further revealed that the main differences in volatile profiles among YW1, YW2, and YW3 were primarily associated with alcohols, alkanes, phenols, esters, and certain heterocyclic compounds (Figure 8). Further, based on OPLS-DA combined with the screening criteria of VIP > 1, FC ≥ 2 or ≤0.5, and *p* < 0.05, differential aroma compounds among groups were identified.

In the comparison between YW1 and YW2, Phenylethyl alcohol (rose-like floral aroma), terpinen-4-ol (sandalwood-like aroma), 2,4,4-trimethyl-2-cyclohexen-1-ol (woody and fruity notes), and safranal (herbal woody aroma) were significantly upregulated in YW1. In contrast, p-xylene (aromatic note), 3,5-dimethylphenol (musty odor), 2-ethyl-1-hexanol (floral note), (+)-α-pinene (woody aroma), hexadecanoic acid methyl ester (fatty note), 1-(1H-pyrrol-2-yl)ethanone (roasted aroma), and 2-undecanone (fruity and fatty notes) were significantly downregulated in YW1.

YW1 was characterized by floral–woody–spicy notes, whereas YW2 was dominated by resinous, fatty, roasted, and phenolic odor characteristics.

In the comparison between YW1 and YW3, hexadecane (FC = 9282.50), phenylethyl alcohol (FC = 691.10; rose-like floral aroma), terpinen-4-ol (FC = 662.42; peppery and sandalwood-like aroma), and 2,4,4-trimethyl-2-cyclohexen-1-ol (FC = 3819.00; woody and fruity notes) were significantly upregulated in YW1.

In contrast, 3,5-dimethylphenol (FC = 0.00; musty odor), hexadecanoic acid methyl ester (FC = 0.00), undecane (FC = 0.01), camphene (FC = 0.05; woody and camphoraceous notes), 1-(1H-pyrrol-2-yl)ethanone (FC = 0.04; roasted aroma), (+)-α-pinene (FC = 0.05; pinewood and resinous notes), tetradecane (FC = 0.01; pinewood and resinous notes), 2-ethyl-1-hexanol (FC = 0.04; floral note), and 1-octen-3-ol (FC = 0.11; mushroom-like odor) were significantly downregulated in YW1.

YW1 was characterized by a cleaner profile dominated by floral, woody, and spicy notes, whereas YW3 exhibited more pronounced fatty–waxy, resinous, roasted, and certain musty or fungal-like odor characteristics.

In the comparison between YW1 and YW3, hexadecane was significantly higher in YW2 than in YW3 (FC = 17,531.00), whereas p-xylene (FC = 0.32), undecane (FC = 0.04), tetradecane (FC = 0.01), hexadecanoic acid methyl ester (FC = 0.03), safranal (FC = 0.07), and 2-tridecanone (FC = 0.02) were significantly downregulated in YW2.

Overall, the aroma evolution of Raw Pu-erh Tea across different storage stages exhibited a clear directional pattern. Early-stage samples were dominated by floral, spicy, and fruity–woody notes. Mid-to-late-stage samples gradually developed resinous, waxy, and matured aroma characteristics. In contrast, high-age samples further evolved into a complex aroma system characterized by the coexistence of herbal-woody, camphoraceous, roasted, and aged notes. These findings, from the perspective of volatile compounds, confirm that the aroma quality of Raw Pu-erh Tea undergoes continuous restructuring and increasing complexity during long-term storage.

### 3.4. Correlation Analysis Between Volatile and Non-Volatile Compounds in Pu-Erh Raw Tea Across Different Storage Years

Within volatile constituents, p-xylene showed a very strong positive correlation with 2-tridecanone (*p* ≤ 0.001) (Figure 9). Phenylethyl alcohol was strongly positively correlated with safranal, 1-octen-3-ol, and hexadecane (all *p* ≤ 0.001). In addition, (+)-α-pinene exhibited a positive correlation with 2-undecanone, camphene with 2,4,4-trimethyl-2-cyclohexen-1-ol, and tetradecane with camphene, indicating that these volatile compounds share a relatively consistent accumulation trend during sample evolution. Meanwhile, clear correlations were also observed between certain volatile compounds and non-volatile quality indices. Free amino acids (FAA) showed positive correlations with 1-octen-3-ol, safranal, phenylethyl alcohol, and 2-ethyl-1-hexanol (r = 0.69, 0.65, 0.58, and 0.57, respectively), but a relatively strong negative correlation with myricetin and theaflavins (TF) (r = −0.44). In addition, quercetin (Que) showed a strong positive correlation with gallocatechin (GC) (r = 0.72), gallic acid (GA) exhibited a moderate positive correlation with luteolin (r = 0.50), and epigallocatechin (EGC) was positively correlated with FAA (r = 0.52). Conversely, quercetin was negatively correlated with terpinen-4-ol and hexadecanoic acid methyl ester (r = −0.63 and −0.56, respectively).

Overall, volatile aroma compounds exhibited a certain degree of coordinated variation. Notably, FAA showed pronounced differential correlations with flavonoids, theaflavins, and several volatile constituents, suggesting that it may serve as a key quality indicator linking aroma formation with the transformation of non-volatile components.

## 4. Discussion

This study systematically elucidated the quality evolution patterns of Raw Pu-erh Tea stored for 1–22 years from the same origin (Yiwu), and identified four stage-dependent transitions (1–4 years, 6–10 years, 14–18 years, and ≥19 years). The results demonstrated that samples stored for approximately 7 years exhibited the optimal sensory quality. A comparable nonlinear pattern was reported by Cheng et al. [25] in Qingzhuan tea, for which taste quality and metabolic profiles showed a parabolic relationship with storage age, with a turning point around 10 years. Although this supports the importance of an intermediate aging period, the different tea type and evaluation methods preclude transferring that numerical optimum to raw Pu-erh tea. Through comprehensive analyses of both non-volatile and volatile constituents, combined with OPLS-DA and correlation analysis, multiple key differential physicochemical indices and aroma-active compounds were identified. These findings further clarify the chemical basis and coordinated transformation mechanisms underlying the characteristic “improving aroma with age” phenomenon of Raw Pu-erh Tea.

During storage, a series of biochemical transformations occur in tea constituents, including the oxidative polymerization of polyphenols, hydrolysis and isomerization of catechins, Maillard reactions between amino acids and sugars, as well as the degradation of lipids and carotenoids [26]. These changes directly contribute to the evolution of sensory quality in Raw Pu-erh Tea: the infusion color gradually deepens from light yellow to orange-red and even brownish-red; the aroma shifts from fresh, floral, and fruity notes toward woody and aged notes; and the taste transitions from strong bitterness and astringency to a mellow, sweet, and smooth profile [27,28].

This study found that Raw Pu-erh Tea during 1–22 years of storage could be divided into four distinct stages, and that physicochemical differences became significantly weaker after 14 years, with internal constituents gradually reaching a relatively stable state. This result is generally consistent with the review by Ma [4], which suggested that the optimal aging period of Raw Pu-erh Tea is approximately 10–20 years; however, the present study further refined the boundaries between the early stage (1–4 years) and the active transformation stage (6–10 years).

The study by Zhou B et al. [6] on quality indicators during Raw Pu-erh Tea storage reported that sensory quality improves after 10 years of storage but begins to decline after 18 years, which is in agreement with the trend observed in this study, where certain quality indices became relatively flat after 19 years. Notably, in this work, cluster analysis of volatile compounds classified samples into three stages (1–10 years, 14–19 years, and 20–22 years), whereas non-volatile constituents were grouped into four stages, suggesting that the evolution of aroma compounds may lag behind the staged differentiation of physicochemical constituents, or may be more directly driven by microbial succession [11]. Chen et al. [29] also observed stage-dependent aroma differentiation in Tuo tea stored for 2–25 years, but their boundaries were 2–4, 5–10, and 13–25 years. The shared feature is a staged rather than uniform change in aroma; the different boundaries indicate that aging classifications depend on the sample collection, product characteristics, and analytical variables. Lv et al. [2], in their review, summarized storage-related quality evolution patterns across different tea types and pointed out that the positive quality development of Raw Pu-erh Tea requires long-term natural aging. This study validates that conclusion using a continuous-year dataset and, for the first time at a 22-year scale, identifies the 14–18-year period as a critical accumulation phase.

This study found that water-extractable substances and soluble sugars reached their peak at 10 years of storage, whereas free amino acids and EGCG increased again in the later stage, while theaflavins and myricetin continuously decreased (*p* < 0.05), and flavonoid glycosides such as rutin showed significant accumulation (*p* < 0.05). This trend is generally consistent with the findings of Ma et al. [8], who reported an initial decrease followed by an increase in water-extractable substances and a continuous decline in tea polyphenols. However, the present study further clarified that 10 years represents a common peak point for both water-extractable substances and soluble sugars, and the secondary increase in EGCG in the late stage (reaching 61.32 mg/g at 22 years) is particularly noteworthy.

Conventional perspectives suggest that ester-type catechins continuously decrease during aging [6]. In contrast, the observed re-increase in EGCG during 19–22 years in this study may be associated with microbially mediated catechin metabolic restructuring. Yang et al. [14], using hyperspectral imaging combined with microbiome analysis, reported that under warm–humid storage conditions, the relative abundance of Bacillus increased significantly (reaching 40.44% at 6 years) and was strongly negatively correlated with the degradation of ester-type catechins. In the present study, under Yiwu storage conditions (relative humidity ≤ 70%, temperature ≤ 25 °C), a relatively mild microbial ecosystem may have been formed. The enrichment of certain bacteria in the mid-to-late stage (e.g., *Paenibacillus* and *Pullulanibacillus*) or changes in the metabolic activity of specific fungi (e.g., *Aspergillus*) may have promoted the regeneration or transformation of catechin-related compounds [12].

In addition, the continuous accumulation of flavonoid glycosides such as rutin, together with the strong positive correlation between flavonoids and gallocatechin (GC) (r = 0.72), suggests a highly coordinated rather than purely competitive relationship between flavonoid and catechin degradation pathways. This finding is consistent with the metabolite network constructed by Ma et al. [11] based on untargeted metabolomics, in which flavonoid and catechin nodes were highly interconnected.

Regarding the dynamic changes in free amino acids (FAA), this study observed a significant increase in FAA content at 21 years, reaching 8.84%, and the fold-change differences between low- and high-age groups showed a decreasing trend with prolonged storage. This finding differs from the report by Liu et al. [7], who observed an overall decline in amino acids during Raw Pu-erh Tea storage, and may reflect a dynamic equilibrium between protein degradation and amino acid consumption during long-term aging. Li L et al. [30], in their study on Pu-erh ripe tea from different production areas, reported that amino acids can accumulate in the late storage stage due to microbial proteolysis, which supports the observations of the present study. In addition, theaflavins in this study reached a peak at 7 years (0.09%) and then continuously declined, which is consistent with the findings of Zhou B et al. [6], who reported a decrease in theaflavins after approximately 10 years of storage. This further suggests that theaflavins may serve as potential marker compounds for mid-stage transformation during Pu-erh tea aging.

For instance, warm and humid environments (such as those in Guangdong Province) can accelerate the hydrolysis of ester-type catechins and promote the accumulation of methoxybenzene-derived aroma compounds, thereby facilitating the formation of aged and woody notes. In contrast, dry and cool environments (such as Kunming) are more conducive to preserving floral and fruity aroma characteristics [13].

This study found that β-ionone (floral aroma) and (E)-2-nonenal (woody aroma) maintained ROAVs greater than 1 across all storage years, forming an “aroma backbone” that persisted throughout the entire aging process. This finding is not contradictory to the report by Li L et al. [30], who observed that 1,2,3-trimethoxybenzene and 1,2,4-trimethoxybenzene increased with storage time, while alcohols and acids decreased. This is because the core backbone compounds identified in the present study belong to different chemical categories, and ROAV reflects odor contribution rather than absolute concentration. This broad transition resembles the replacement of fresh, fruity, and floral notes by stale, woody, and herbal attributes reported for stored Tuo tea [29]. This is consistent with the sensory evolution observed in the present study, which showed a transition from floral and fruity notes to woody notes and eventually to aged aroma.

More importantly, this study found that floral- and fruity-related compounds (e.g., phenylethyl alcohol and terpinen-4-ol) were significantly enriched in the early stage, whereas resinous- and woody-related compounds (e.g., camphene, (+)-α-pinene, and hexadecanoic acid methyl ester) increased significantly in the mid-to-late stages. Zhao et al. [12], using a combined volatilomics and microbiomics approach, confirmed that *Aspergillus* became a dominant fungal genus during long-term storage and was positively correlated with aged aroma compounds such as 1,2,3-trimethoxybenzene and 1,2,4-trimethoxybenzene. This is highly consistent with the accumulation of methoxybenzene compounds observed in high-age samples in the present study.

In addition, the present study identified up to 112 volatile compounds and 39 key aroma-active compounds, both of which increased with storage time, indicating that aroma complexity progressively increased rather than simply declining during aging. This finding supports the conclusion of Lv et al. [2] that tea storage does not simply lead to an increase in total aroma intensity, but rather to an optimization of aroma quality and a transformation of aroma types.

One of the key innovations of this study lies in revealing the significant correlations between non-volatile and volatile constituents. Free amino acids (FAA) showed significant positive correlations with 1-octen-3-ol, safranal, phenylethyl alcohol, and other aroma compounds (r = 0.57–0.69), suggesting that amino acids may participate in the formation of key aroma compounds through Maillard reactions or microbial metabolic pathways. Although this mechanism has been reported in tea processing systems [31], the present study provides the first quantitative validation based on long-term aging datasets of Raw Pu-erh Tea. Meanwhile, FAA exhibited negative correlations with myricetin and theaflavins, indicating that amino acid accumulation may be competitively or consumptively linked with polyphenol oxidation and flavonoid transformation pathways. This provides a plausible explanation for the observed phenomenon of “re-increased amino acids in the late stage accompanied by decreased theaflavins.” In addition, the positive correlations between quercetin and gallocatechin (GC), as well as between gallic acid and luteolin, further support the highly coupled nature of flavonoid–catechin transformation during oxidative degradation pathways. Ma et al. [11], by constructing a metabolic network, reported that with increasing storage time, both the number of nodes and connections in the metabolite network increased significantly, and network modularity was enhanced, while core metabolites (including flavonoids and amino acids) played central roles within the network. The correlation analysis in the present study provides further evidence from another perspective supporting the coordinated evolutionary behavior of this metabolic network during Pu-erh tea aging.

Although microbial communities were not directly measured in this study, numerous investigations have confirmed their central role in the aging process of Raw Pu-erh Tea. Ma [4] summarized that maintaining temperature at approximately 25 °C and controlling relative humidity below 75% are beneficial for quality improvement, and that microbial communities (e.g., *Achromobacter* and *Corynebacterium*) show significant positive correlations with aroma and polyphenolic indices. Yang et al. [14], using 16S rRNA sequencing, reported that under warm–humid storage conditions, the relative abundance of *Bacillus* increased significantly with storage time and was closely associated with the degradation of tea polyphenols and ester-type catechins. In contrast, microbial succession proceeded more slowly under dry and cool conditions. In the present study, the storage conditions (relative humidity ≤ 70%, temperature ≤ 25 °C) correspond to a relatively dry environment, which may explain why the emergence of aged aroma characteristics occurred later (6–10 years) compared with humid regions such as Guangdong (approximately 3–5 years) [13].

Zhao et al. [12] further revealed a microbial succession pattern from *Blastobotrys* to Aspergillus in fungal communities, and from *Bacillus* to *Paenibacillus* and *Pullulanibacillus* in bacterial communities. These microorganisms can secrete enzymes such as polyphenol oxidase, peroxidase, and cellulase, thereby driving catechin degradation and transformation, the formation of methoxybenzene compounds, and the reconstruction of aroma profiles. The observed phenomena in this study, including the secondary increase in EGCG and enrichment of non-esterified catechins during 14–18 years, may be associated with enhanced metabolic activity of specific microbial taxa in the mid-to-late storage stage, although this hypothesis requires further validation through metagenomic approaches.

Moreover, the integration of hyperspectral imaging and machine learning approaches [14] may enable non-destructive and rapid discrimination of storage age, providing a promising tool for quality control in the Pu-erh tea industry. It should be emphasized that microbial communities were not directly characterized in the present study. Therefore, the potential microbial contributions discussed above are proposed based on previous literature and should be regarded as possible mechanisms rather than direct evidence from the present dataset.

## 5. Conclusions

In conclusion, this study systematically characterized the sensory and chemical changes in Raw Pu-erh Tea during 1–22 years of storage. The results showed that Raw Pu-erh Tea exhibited clear stage-related quality evolution during long-term storage, with sensory characteristics gradually shifting from fresh, bitter, and astringent notes to a more mellow taste, deeper liquor color, and more pronounced aged aroma. Based on the changes in non-volatile components, four storage stages were identified, while volatile profiles further reflected distinct aroma evolution patterns across storage years.

Several non-volatile components, including water extracts, free amino acids, soluble sugars, catechins, gallic acid, theaflavins, thearubigins, and theabrownins, showed marked changes during storage and may serve as useful chemical indicators for evaluating quality evolution. In terms of aroma, β-ionone, (E)-2-nonenal, 2,4,4-trimethyl-2-cyclohexen-1-ol, terpinen-4-ol, 4-ethylphenol, camphene, cedrol, and borneol were identified as important aroma-active compounds contributing to floral, woody, spicy, resinous, and aged aroma characteristics. These changes were consistent with the observed sensory transformation during storage.

The continuous 1–22-year sample set provides a useful basis for understanding the long-term quality evolution of Raw Pu-erh Tea. The identified non-volatile and volatile markers may contribute to storage-stage classification, quality control, and age authentication of Raw Pu-erh Tea. However, the relationships observed in this study are mainly based on correlation and compositional analyses; therefore, further targeted experiments are needed to verify the formation pathways and causal roles of these potential markers during storage.

## Figures and Tables

**Figure 1:**
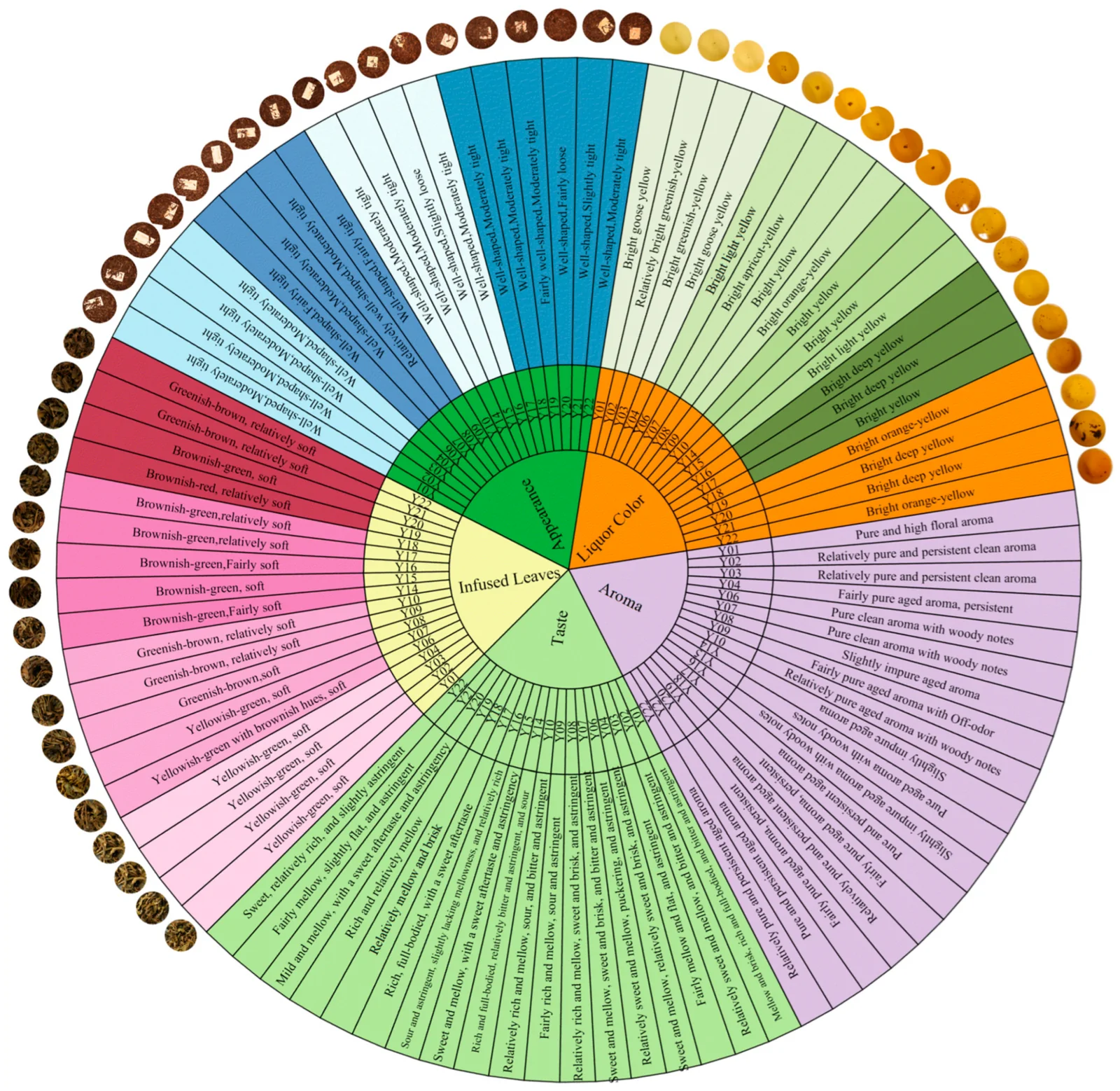
Sensory evaluation flavor wheel of Raw Pu-erh Tea from different storage years.

**Figure 2:**
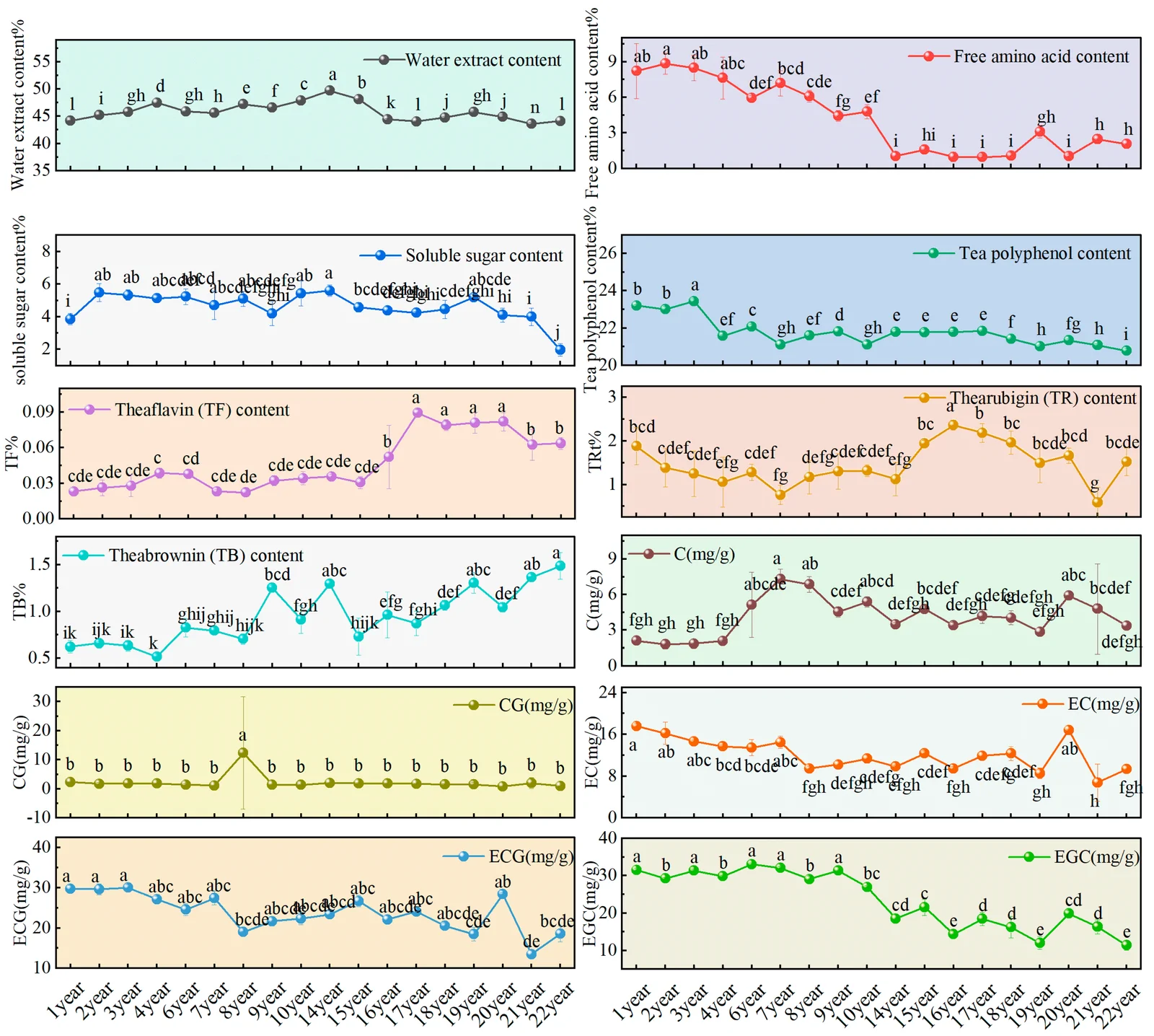
Contents of major chemical constituents in Raw Pu-erh Tea with different storage years. (catechin (C), catechin gallate (CG), epicatechin (EC), epicatechin gallate (ECG), epigallocatechin (EGC), and epigallocatechin gallate (EGCG), Different lowercase letters indicate significant differences among storage years for the same compoun).

**Figure 3:**
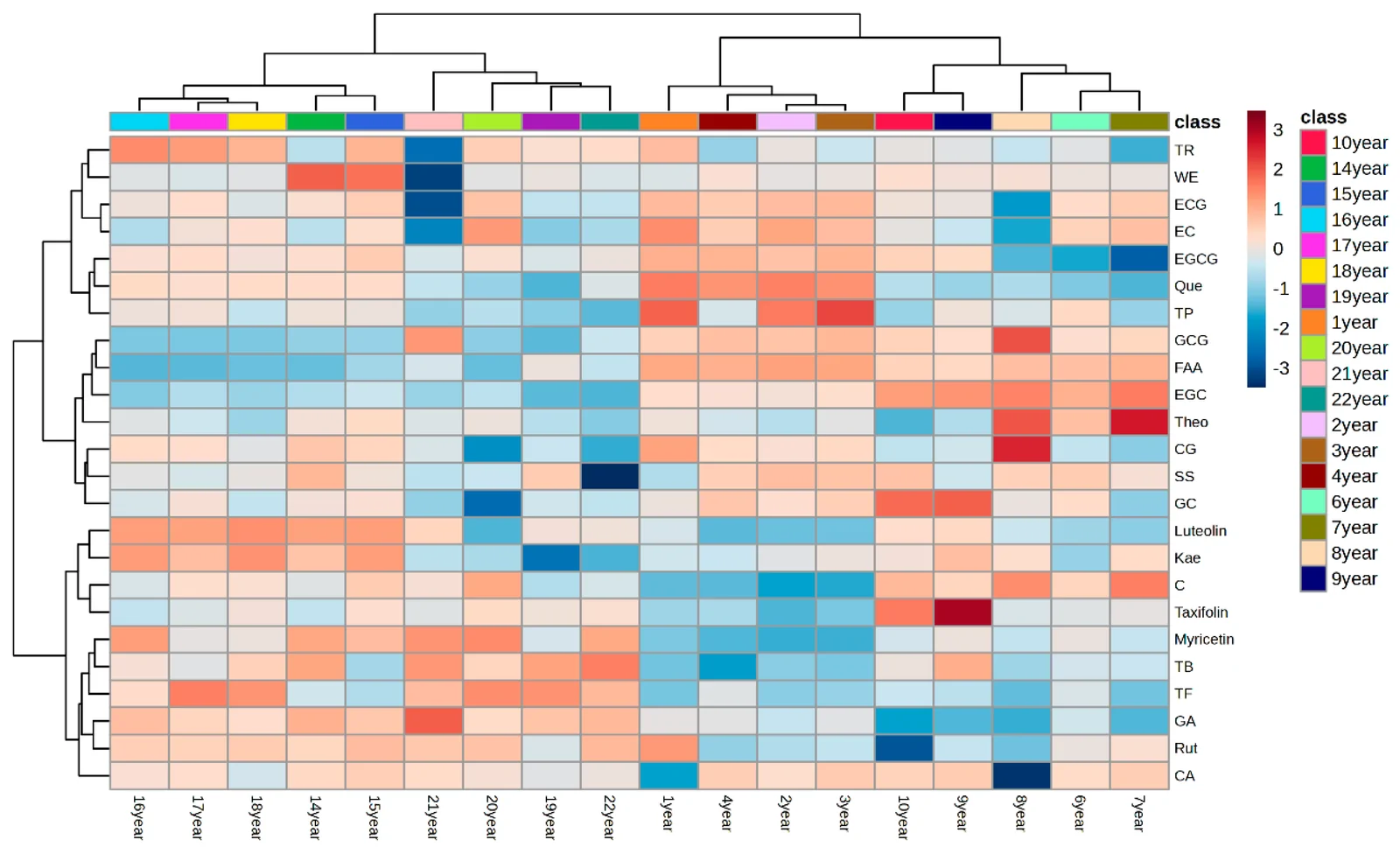
Cluster heatmap of 24 physicochemical indices of Pu-erh raw tea across different storage years.

**Figure 4:**
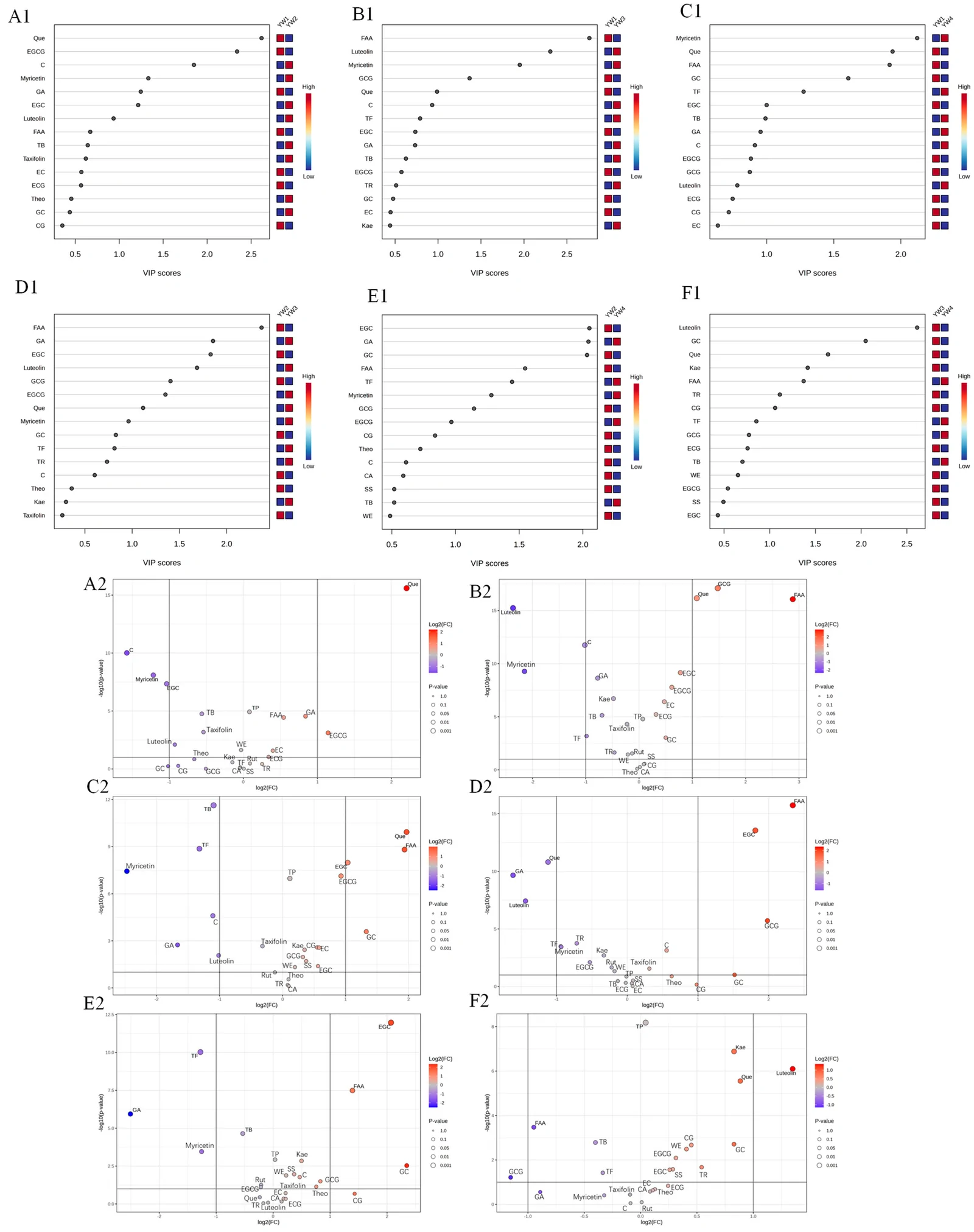
Variable importance in projection (VIP) scores and volcano plots of non-volatile compounds across the four groups. (**A1**–**F1**) VIP scores for W1 vs. W2, W1 vs. W3, W1 vs. W4, W2 vs. W3, W2 vs. W4, and W3 vs. W4, respectively. The colored squares indicate the relative levels of each compound in the two groups shown above each panel. (**A2**–**F2**) Corresponding volcano plots for the same six comparisons, respectively.

**Figure 5:**
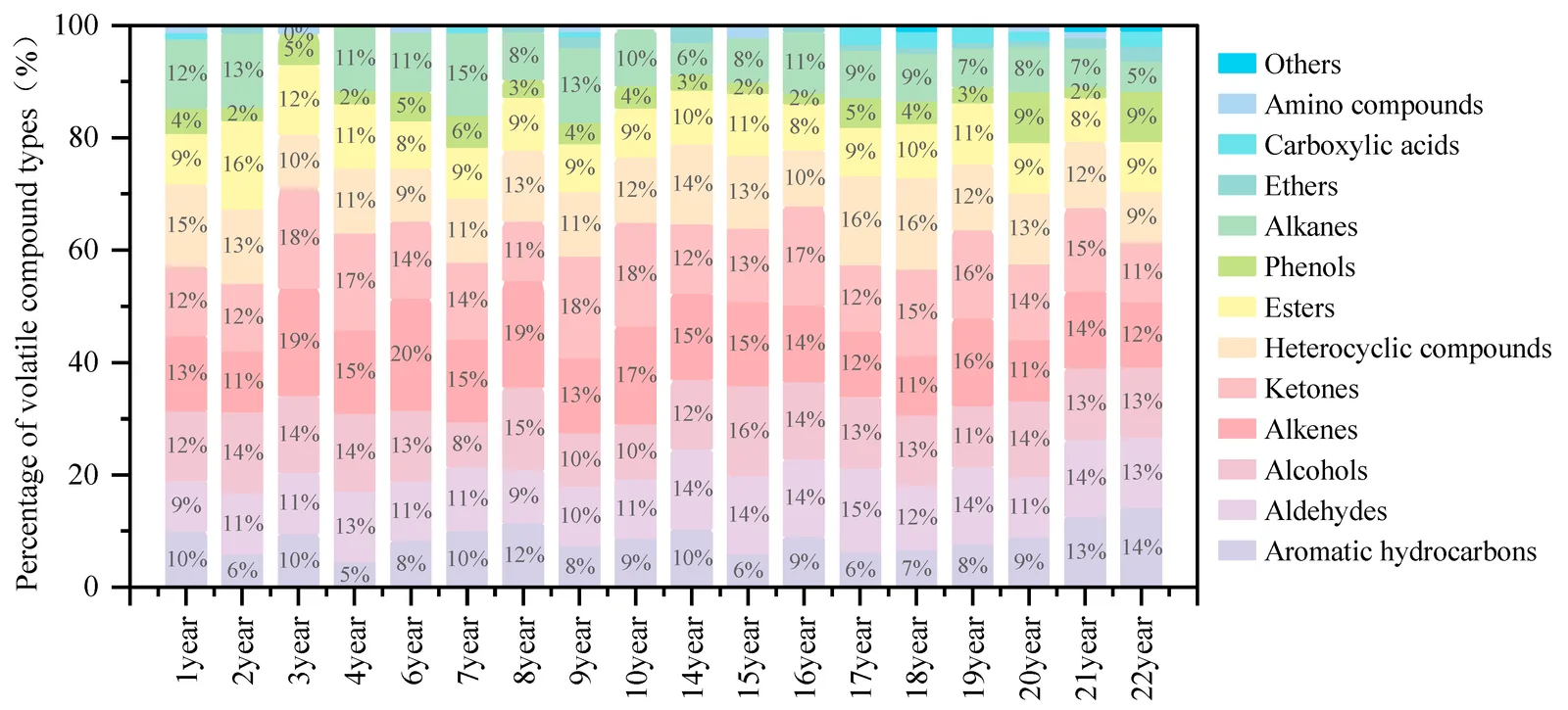
Percentage stacked bar chart of volatile compound classes in Pu-erh raw tea across different storage years.

**Figure 6:**
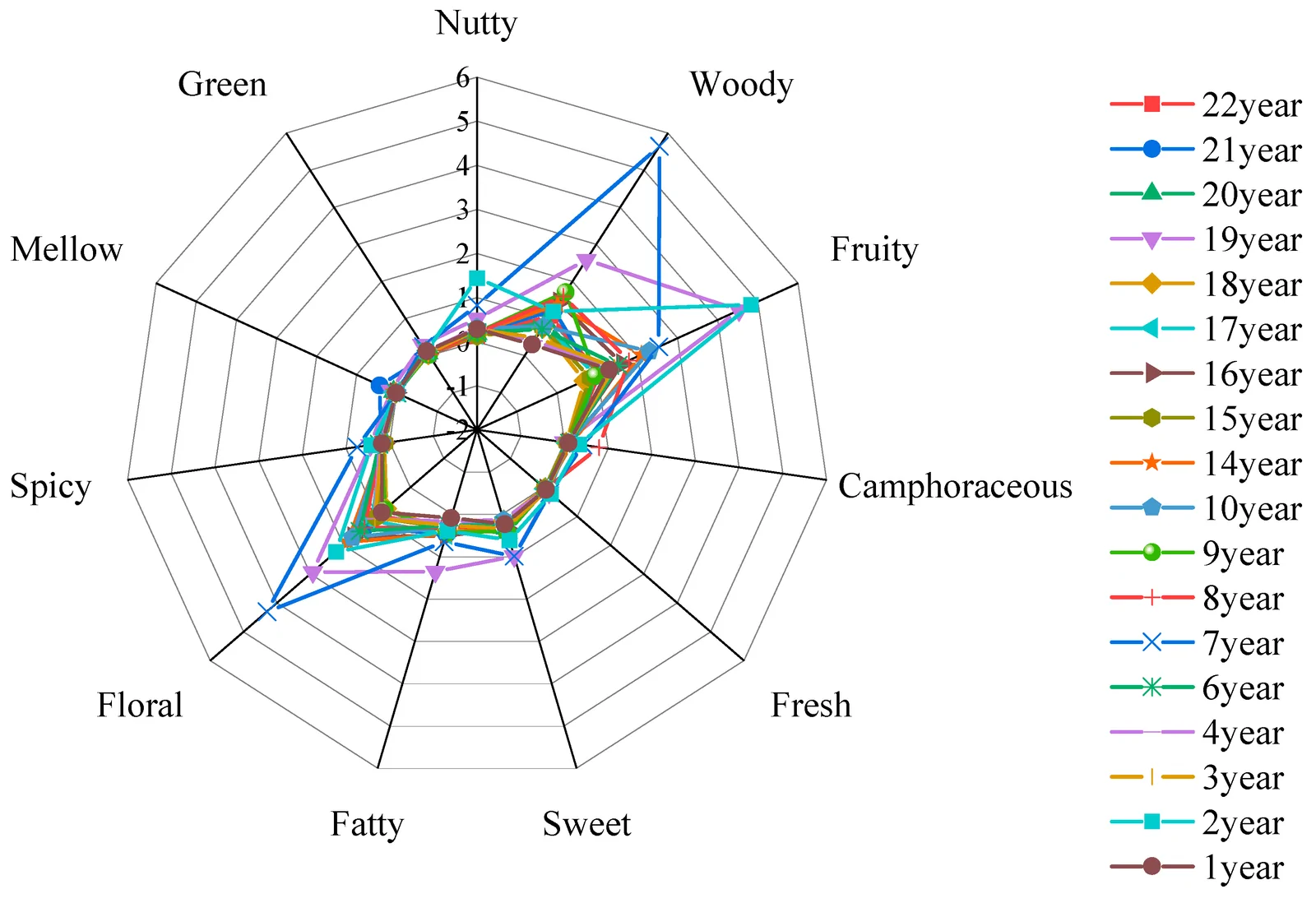
Radar chart of aroma profile types of volatile compounds in Pu-erh raw tea across different storage years.

**Figure 7:**
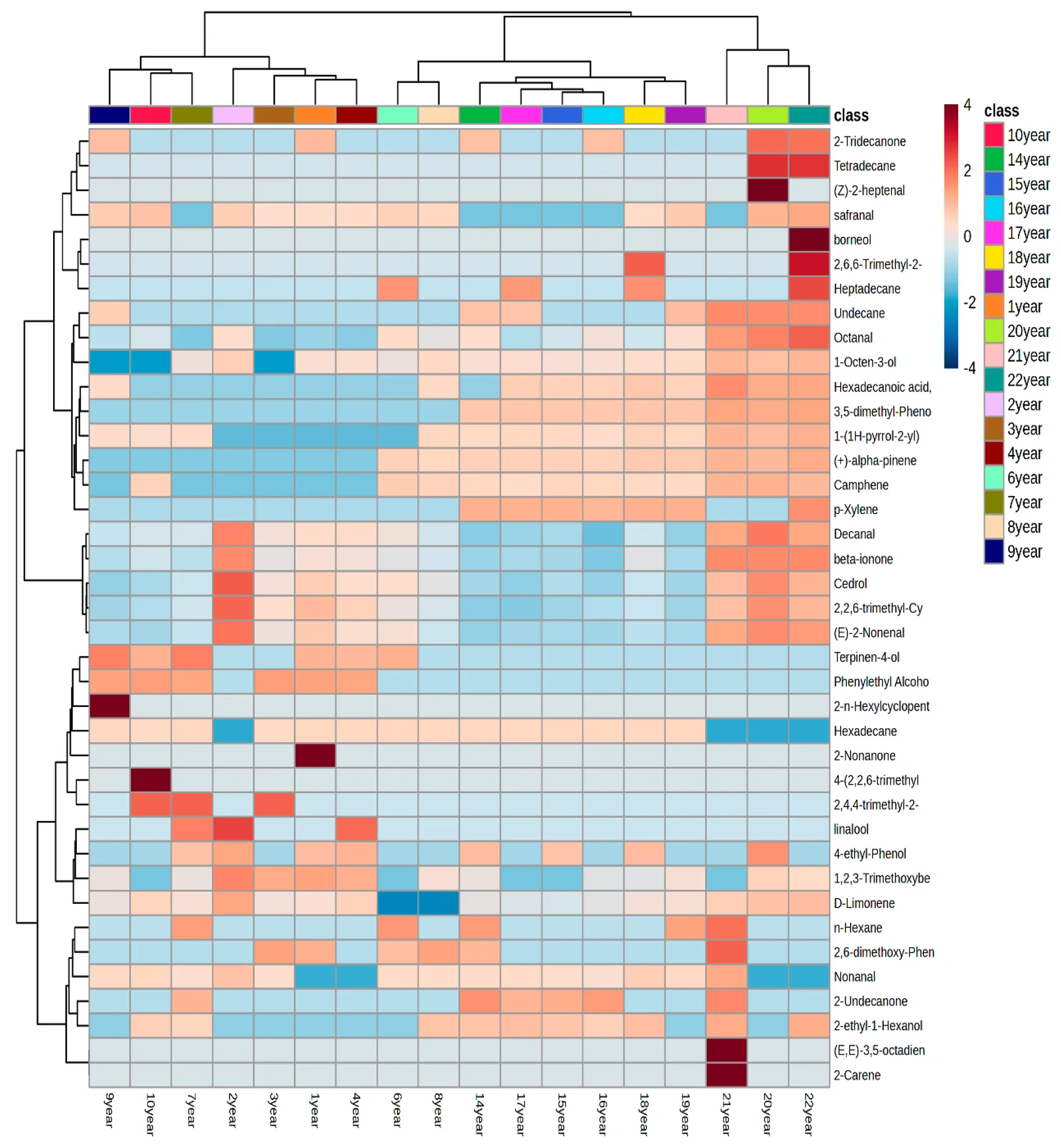
Cluster heatmap of volatile compounds with ROAV > 1 in Pu-erh raw tea across different storage years.

**Figure 8:**
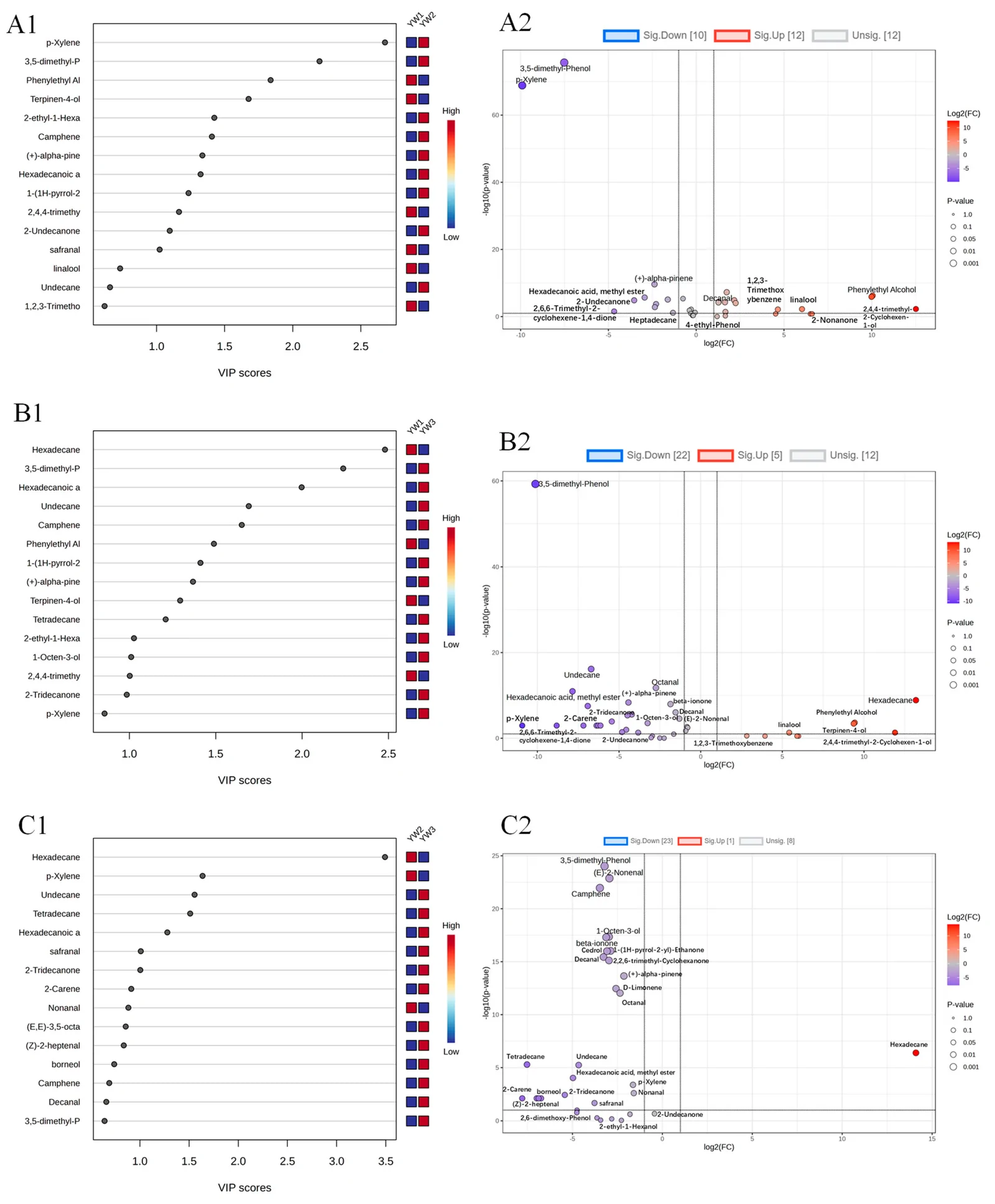
Variable importance in projection (VIP) scores and volcano plots of volatile compounds among the three aroma stages. (**A1**,**A2**) YW1 vs. YW2; (**B1**,**B2**) YW1 vs. YW3; (**C1**,**C2**) YW2 vs. YW3.

**Figure 9:**
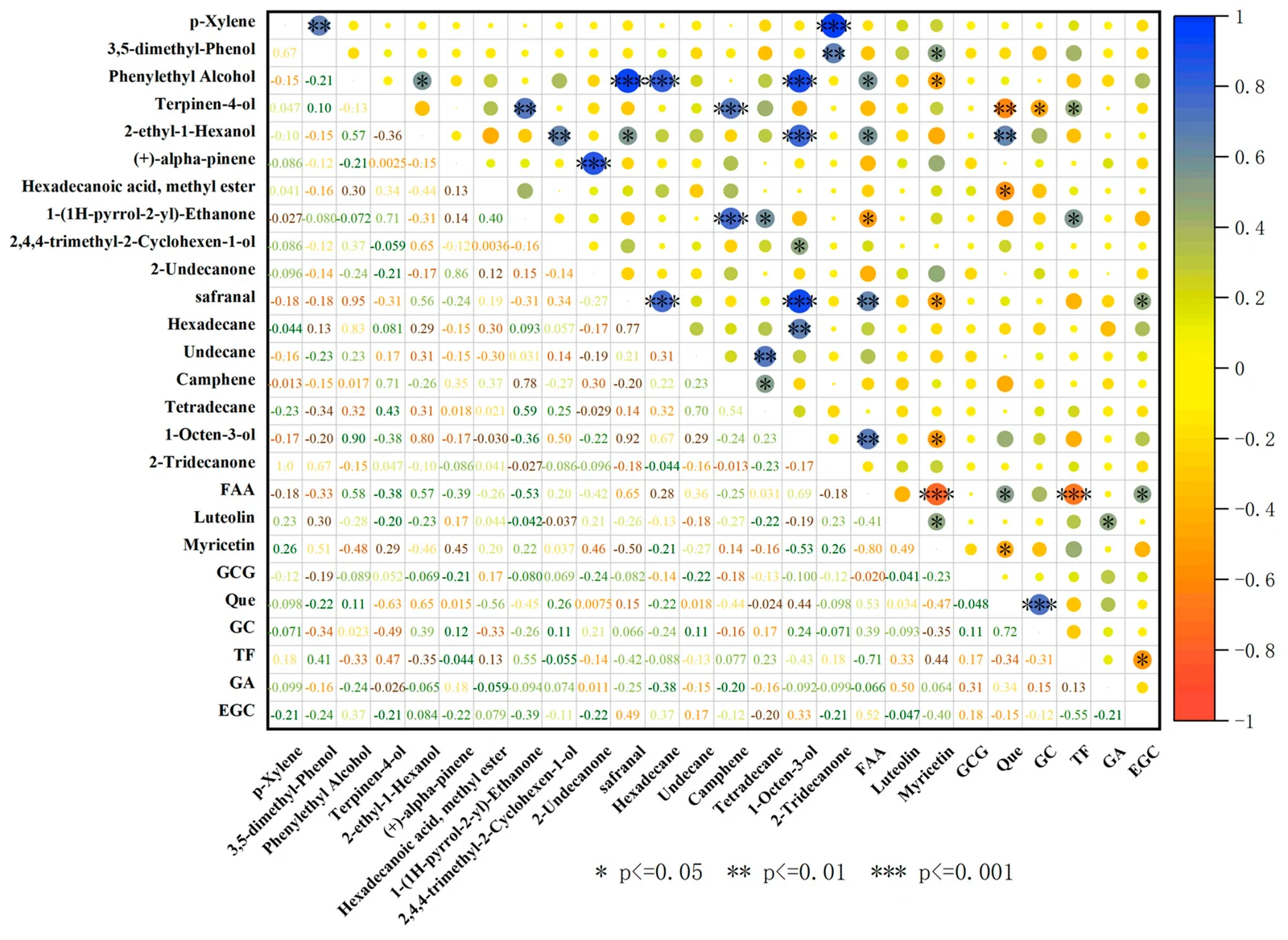
Correlation heatmap of key differential non-volatile and volatile compounds in Pu-erh raw tea across different storage years.

**Table 1:** Experimental tea sample information.

Sample ID	Production Year	Aging Time (Years)
1 year	2023	1 year
2 year	2022	2 years
3 year	2021	3 years
4 year	2020	4 years
6 year	2018	6 years
7 year	2017	7 years
8 year	2016	8 years
9 year	2015	9 years
10 year	2014	10 years
14 year	2010	14 years
15 year	2009	15 years
16 year	2008	16 years
17 year	2007	17 years
18 year	2006	18 years
19 year	2005	19 years
20 year	2004	20 years
21 year	2003	21 years
22 year	2002	22 years

Note: All tea samples were produced in Yiwu, Xishuangbanna, Yunnan, China, by Yunnan Six Famous Tea Mountains Co., Ltd., and were analyzed in 2024.

## Data Availability

The original contributions presented in this study are included in the article materials. Further inquiries can be directed to the corresponding authors.
